# Editorial: More than a “formulas problem”: IOL power calculation and biometry in the era of “refractive cataract”

**DOI:** 10.3389/fnins.2025.1664035

**Published:** 2025-08-27

**Authors:** Ferdinando Cione, Nicola Rosa, Oleksiy Voytsekhivskyy

**Affiliations:** ^1^Ophthalmological Unit, Department of Medicine, Surgery and Dentistry, Scuola Medica Salernitana, University of Salerno, Salerno, Italy; ^2^AOU San Giovanni di Dio e Ruggi D'Aragona, Salerno, Italy; ^3^Kyiv Clinical Ophthalmology Hospital “Eye Microsurgery Center”, Kyiv, Ukraine

**Keywords:** IOL power calculation, surgical training, IOL material, myopia, implantable collamer lens; cognitive function, visual function

Cataract and refractive surgeries are some of the globally most performed ophthalmic interventions, with rapidly evolving implications, that extend beyond visual rehabilitation. Recent studies have highlighted their impact not only on quality of vision, but also on cognitive functions and neural processing. These innovative outcomes have been addressed in this Research Topic, that aims to change the way to see cataract and refractive surgery procedures: not only a “recovery vision” procedure, but quality of life-change surgeries. For this reason, it should be pointed out that new parameters play a role when the clinician has to plan a cataract or a refractive surgery.

For example, in studies investigating intraocular lens (IOL) power calculation, refractive prediction error (PE) is generally considered the primary outcome; therefore, papers predominantly focus on formula accuracy (Voytsekhivskyy et al., 2025; Stopyra et al., 2025), biometric parameter analysis (Cione et al., 2024a; Tutchenko et al., 2024; De Bernardo et al., 2022), or lens constants optimization (Cione et al., 2024b), with particular emphasis on challenging eyes, such as post-refractive surgery eyes (Cione et al., 2023).

However, it is equally important to evaluate the quality of the surgical technique itself and the level of surgical training, as these factors can significantly influence refractive outcomes and the overall success of cataract surgery. A prospective cohort study carried out in Ethiopia by Atoma Gelalcha et al. assessed postoperative visual outcomes in 341 cataract patients. Remarkably, surgical residents achieved outcomes comparable to senior ophthalmologists, with 74.2% of patients reaching a best corrected visual acuity (BCVA) ≥ 6/18 and 54.8% achieving 6/12. These findings underscore the importance of structured surgical training in achieving global eye health targets.

Another key domain, which also plays a crucial role in neuroadaptation, is the analysis of materials used in IOLs. These materials significantly affect not only optical performance and biocompatibility, but also postoperative complications, such as posterior capsule opacification, glistenings, and dysphotopsias. Emerging advanced polymeric materials (e.g., hydrogels, UV-blocking, nanostructured coatings) have been investigated for their potential to reduce optical scatter, enhance biocompatibility, and support personalized visual outcomes (Wu et al., 2024). In the era of “refractive cataract,” the so-called “premium” IOLs play a crucial role and consequently, the choice of the right material is essential to avoid halos, glares and other types of dysphotopsias. Cabanás et al., in a case-based study, identified surface whitening (nanoglistenings) on hydrophobic trifocal IOL, correlated with visual disturbances such as reduced contrast sensitivity and glare. These findings raise critical considerations for IOL material longevity and optical performance, emphasizing the need for post-market surveillance: a rigorous evaluation of IOL materials and optical performance to safeguard long-term visual quality is required.

An important role is played by the analysis of refractive and surgical outcomes in myopic eyes, given the well-documented global increase in the prevalence of myopia (Cione et al., 2024a). Two studies included in this Research Topic investigate various aspects related to visual quality in myopic eyes. Wu and Leung, combining psychophysical contrast sensitivity tests with steady-state visual evoked potentials, revealed orientation-specific neural processing deficits in individuals with high myopic astigmatism. These findings suggest that optical and neural anisotropies together contribute to visual quality impairments, providing a foundation for optimizing refractive correction strategies. In addition, Nie et al. proposed a review that synthesized evidence on subjective and objective visual quality following implantable collamer lens (ICL) implantation for myopia. Key findings include transient increases in higher-order aberrations postoperatively, improved optical metrics within 1–3 months, and high prevalence of halos and glare. Quality optimization strategies include pharmacologic pupil modulation and refined surgical techniques. The authors highlight the need for long-term, standardized studies to optimize ICL outcomes.

As noted in the preceding paragraphs, PE alone no longer represent the sole parameter for evaluating postoperative outcomes in cataract surgery studies. A new analytical framework has emerged, focusing on the improvement of cognitive parameters following cataract surgery. Zhao et al. demonstrated that cataract surgery significantly improves both cognitive performance and visual function (stereopsis, simultanagnosia) in elderly individuals. Particularly, bilateral surgery resulted in greater cognitive benefits, particularly among those with mild cognitive impairment, highlighting the procedure's role in maintaining neurocognitive health. Anyway, with the increasing use of multifocal and extended depth-of-focus (EDOF) IOLs, accurate evaluation of PE still plays a crucial role. (Bellucci et al. 2023) emphasized how systemic errors in automated refraction could occur in case of multifocal and EDOF IOLs implants. In detail, autorefractors tend to overestimate myopia in such IOLs categories, probably due to the amount of diffracted light. This is very relevant: in fact, an emmetropic or slightly hyperopic automated refraction may falsely suggest correct IOL power, while the multifocal component remains ineffective. Therefore, information about the expected myopic error should be included in the preoperative statement of multifocal and EDOF IOLs (Bellucci et al., 2023). The expanding role of cataract and refractive surgery in improving not only vision but also cognitive function and neural processing should be underlined.

In summary, this Research Topic illustrates the multidimensional benefits and complexities of modern cataract and refractive surgery. Refractive PE remains a major target (Voytsekhivskyy et al., 2025; Stopyra et al., 2025; Cione et al., 2024a), and the role of reliable biometry is still predominant (Cione et al., 2024a; Tutchenko et al., 2024; De Bernardo et al., 2022), but the planning of a surgical intervention requires the evaluation of additional parameters, which are essential to achieve surgical success:

- Taking into consideration that cataract and refractive surgery can improve not only vision but also cognitive function and neural processing;
- The importance of surgical training programs;
- The need for rigorous evaluation of IOL materials and optical performance to safeguard long-term visual quality;
- The emerging relevance of neural biomarkers for characterizing and correcting refractive deficits.

In conclusion, this Research Topic has demonstrated that cataract and refractive surgeries are not only a “formula problem” or a “refractive problem” but a fascinating field that can actively influence quality of life of patients.
